# Effects of Different Levels of Drought Stress in *Ficus* Plants on the Life History and Population Growth of *Perina nuda* (*Lepidoptera*: *Lymantriidae*): An Age-Stage, Two-Sex Life Table Analysis

**DOI:** 10.3390/insects17010048

**Published:** 2025-12-30

**Authors:** Changqi Chen, Yunfang Guan, Yan Wang, Ying Zhang, Zhu Liu, Yana Zhou, Zongbo Li, Yuan Zhang

**Affiliations:** 1 Key Laboratory of Forest Disaster Warning and Control of Yunnan Province, College of Forestry, Southwest Forestry University, Kunming 650224, China; chen_cq@swfu.edu.cn (C.C.); g_yunfang@swfu.edu.cn (Y.G.); yan_wang@swfu.edu.cn (Y.W.); zhangying@swfu.edu.cn (Y.Z.); liuzhu@swfu.edu.cn (Z.L.); zhouyana@swfu.edu.cn (Y.Z.); lizb@swfu.edu.cn (Z.L.); 2 College of Forestry, Southwest Forestry University, Kunming 650224, China

**Keywords:** drought stress, *Perina nuda*, *Ficus macrocarpa*, age-stage two-sex life table, population parameters, fitness

## Abstract

Under the background of global climate change, frequent drought events have significantly impacted plant–insect interaction. This study focuses on *Ficus microcarpa*, an important landscaping and urban greening tree species in tropical and subtropical regions, and its primary herbivorous pest, *Perina nuda*, by applying the age-stage, two-sex life table theory to systematically evaluate the effects on the life history traits and population dynamics of *P. nuda* reared on *F. microcarpa* subjected to different levels of drought stress. The results demonstrated that reared on drought-stressed *F. microcarpa* significantly altered multiple life history traits of *P. nuda*. All drought treatments significantly shortened the larval development period. Under both light and severe drought conditions, adult lifespan was prolonged, the total pre-oviposition period was reduced, fecundity per female increased, and generation time (*T*) was shortened. However, significant increases in pupal weight, intrinsic rate of increase (*r*), and finite rate of increase (*λ*) were observed only under light drought stress. The population prediction results indicate that both light and severe drought stress lead to obviously higher population growth rates and larger population sizes at 200 days compared to the control group. These findings suggest that the population fitness of *P. nuda* is enhanced under light and severe drought stress, potentially increasing the probability of pest outbreaks. This study provides an important theoretical basis and practical advice on forecasting population dynamics and implementing integrated management strategies for *P. nuda* in the context of climate change.

## Simple Summary

Based on the age-stage, two-sex life table theory, this study systematically investigated the effects of reared on *Ficus microcarpa* subjected to different levels of drought stress on the life history traits and population dynamics of *Perina nuda*. The research findings demonstrate that the effects of different levels of drought stress on *P. nuda* exhibit a nonlinear pattern. Under light drought stress, the fitness of this pest reaches its peak, manifested by optimal levels across key parameters including development, reproduction, and population growth. In contrast, moderate drought stress had a relatively weak effect; while severe drought stress promoted some life history parameters, its overall impact was weaker than that of light drought stress. The population prediction results indicate that both the growth rate and population size of *P. nuda* under light and severe drought stress are obviously higher than the control group. These findings suggested that light and severe drought stress enhanced the suitability of *F. microcarpa* as a host plant, thereby potentially increasing the risk of pest outbreaks. This study provides a critical scientific foundation for predicting population dynamics and implementing ecological management strategies for *P. nuda* in the context of climate warming.

## 1. Introduction

Over the past century, driven by the intensification of greenhouse gas emissions and the gradual degradation of the ecological environment, global drought events have shown a significant increase in frequency, severity, and spatial extent [1,2]. Geographically, the impacts of drought are widespread: around one-third of the world’s land surface is located in arid or semi-arid regions, and periodic droughts often occur in the remaining areas [3]. In China, the problem of drought is extremely severe, as arid and semi-arid regions make up nearly half of the country’s total land area [4]. Multiple studies suggest that unless greenhouse gas emissions are effectively curbed, drought events are likely to become more frequent and intense globally in the next few decades, this will pose substantial threats to the stability of ecosystems and the sustainable development of agriculture and forestry [5,6]. Compared with mobile animal populations, plants are especially susceptible to drought stress because of their immobility, which restricts their capacity to avoid unfavorable environmental conditions [7,8,9].

Previous studies have demonstrated that drought stress not only directly hinders plant growth and development but may also indirectly affect the interactions between plants and herbivorous insects by modifying key physiological and biochemical traits [10]. For instance, drought stress can modify the content and composition of plant defense enzyme activities and trigger plant defense hormone signaling pathways. This, in turn, impacts the life history traits, including growth, development, and reproduction, of pests, and further affects the population dynamics of herbivorous pests [11,12,13]. However, the dynamic changes in plant defense substances under drought stress may have significantly different effects on different insect species. For example, it has been demonstrated that mild drought stress can elevate the amino acid content in wheat (*Triticum aestivum*), thus facilitating the population growth of *Sitobion avenae* [14]. Conversely, when the peach potato aphid (*Myzus persicae*) feeds on potato plants (*Solanum tuberosum*) under drought stress, its growth and development are impeded, resulting in a decreased population growth rate [15].

Currently, research on the population dynamics of herbivorous insects reared on host plants under drought stress remains limited, especially in terms of systematic quantitative analyses of key life history parameters such as development rate, survival rate, and fecundity. As a key plant group in the tropical and subtropical regions of the world, *Ficus* plants play an important role in maintaining ecosystem balance and promoting ecological restoration [16]. Moreover, *Ficus* species are among the most extensively utilized ornamental plants in southern China and serve a significant function in urban landscape planning. Previous studies have demonstrated that *Ficus* plants exhibit a high degree of susceptibility to a diverse array of pests, such as *Perina nuda*, *Phauda flammans* and *Ocinara varians* [17,18,19]. Among these pests, *P. nuda* is widely acknowledged as one of the most devastating pests that afflict *Ficus* plants. This particular pest belongs to the order *Lepidoptera*, the family *Lymantriidae*, and the genus *Perina* [20]. The larvae of this pest are capable of devouring substantial amounts of *Ficus* leaves, which can even result in the complete defoliation of the entire tree. Periodic outbreaks of *P. nuda* in southern regions across China, such as Yunnan, Guangdong, and Fujian provinces, have severely hampered the growth and development of *Ficus* plants, presenting a grave threat to urban greenery and landscaping [21,22]. Nevertheless, there are no reports regarding the life-history traits and population dynamics of *P. nuda* reared on *Ficus microcarpa* under different levels of drought stress. Investigating the interaction mechanisms between *Ficus* plants and herbivorous pests under drought stress is conducive to the effective conservation and management of *Ficus* plants and deepens our understanding of plant–pest interactions in the context of global environmental change.

In integrated pest management and population dynamics prediction, the age-stage, two-sex life table is an important tool in the study of insect population ecology [23]. This tool can systematically evaluate the influence of biotic and abiotic factors, including the environment and food, on the growth and development, reproductive capacity, and population parameters of insects [24,25,26]. It has been extensively utilized in the research of host plant–pest interactions. However, few studies have employed the age-stage, two-sex life table to investigate herbivorous insects on host plants under adverse environmental conditions.

Therefore, based on our prior observations and existing research, we propose the following scientific hypothesis: drought stress in *Ficus* plants may influence the life history traits and population dynamics of *P. nuda.* In order to verify this scientific hypothesis, our study uses *F. microcarpa*, a common urban greening tree species in southern China, and its herbivorous pest *P. nuda* as the research subjects. By using the life table analysis method, we systematically assessed the impact of different levels of drought stress on *F. microcarpa* on the growth, development, and reproductive characteristics of *P. nuda.* By quantifying key population parameters such as developmental duration, survival rate, and fecundity, the population dynamics of *P. nuda* can be revealed. The research findings can offer a scientific foundation for the prediction of *P. nuda* and the formulation of control strategies under the context of climate change. Moreover, it makes significant contributions to the conservation of *Ficus* species in tropical rainforests and provides substantial support for the management of urban green spaces.

## 2. Materials and Methods

### 2.1. Rearing of Insects

Larvae of *P. nuda* were collected from Xishuangbanna Tropical Botanical Garden, Chinese Academy of Sciences, Kunming, Yunnan Province, China, and placed in an insect box (diameter = 8.4 cm, height = 12 cm). After being transported back to the laboratory, they were reared in an artificial climate chamber (Boxun BIC-400, Shanghai, China) with a photoperiod of 14:10 h (light/dark), at a temperature of 25 ± 1 °C and a relative humidity of 50%. To ensure the consistency of the materials used in the experiment, *F. macrocarpa* leaves were employed as the rearing medium in the laboratory, and newly hatched larvae obtained after multiple generations of laboratory rearing were used as the test insect source.

### 2.2. Rearing of Plants

The *F. microcarpa* plants used in the experiment were artificially cultivated. Seeds were collected from mature figs of 3 healthy fig tree at the Xishuangbanna Tropical Botanical Garden, Chinese Academy of Sciences, Yunnan Province, China. These seeds were sown in plastic pots filled with a substrate mixture that was optimized based on preliminary experiments (peat:vermiculite:perlite = 10:10:1, by mass). To maintain the optimal growth conditions, the soil was irrigated every three days with a nutrient solution containing nitrogen, phosphorus, and potassium fertilizers (N:P:K = 1:3:2). Two-year-old *F. microcarpa* plants that exhibited uniform growth and healthy morphological characteristics were selected for subsequent experiments, with an average height of approximately 60 cm. After the experiment was initiated, watering was halted for the drought-stressed group, enabling the soil moisture to gradually decline to the pre-determined drought levels. In contrast, the control group was provided with regular irrigation throughout the experimental period. According to previous research and preliminary experimental findings. The soil moisture levels of each treatment group were as follows: in the control group (with normal water supply, CK), the soil moisture content was maintained at 75–80%; under light drought stress (LD), it was 55–60%; under moderate drought stress (MD), it was 40–45%; and under severe drought stress (SD), it was 30–35% [27]. Each treatment was composed of 20 replicate pots. Once the target drought levels were reached, the potted plants were weighed and their initial masses were recorded. To keep the soil moisture content within the specified treatment ranges, the plants were weighed daily in the evening using a portable electronic balance. Then, distilled water was added to each pot as needed to restore the pre-determined mass. The drought treatment lasted for one month, after which *P. nuda* larvae were transferred to *F. macrocarpa* plants, and the designated soil moisture levels for each treatment group were maintained until the end of the experiment [28].

### 2.3. Life Table Study of P. nuda

Egg masses that were laid on the same day were collected and placed in plastic containers (25 × 15 × 8 cm) lined with filter paper. Once the eggs hatched, the newly hatched larvae were gently and carefully transferred into mesh bags (long = 15 cm, wide = 10 cm) using a soft-bristled brush. A single, newly hatched larva was placed in each mesh bag and each bag was attached to a potted *F. microcarpa* plant that underwent different drought stress treatments. Specifically, the bags were attached to the 3rd–7th fully expanded leaves of each plant. Each treatment group consisted of 30 replicates. After the introduction of larvae, larval feces were removed daily between 16:00 and 18:00, and the survival, growth, and development of the were regularly monitored. Once pupation commences, newly formed pupae were collected and weighed using an electronic balance (Ohaus Corporation, Montville, NJ, USA). The pupae were inspected daily until the adult emerged. Newly emerged male and female adults from the same treatment group were paired and housed in plastic cylindrical containers (diameter = 8.4 cm, height = 12 cm). In cases where a male adult was not available during the pairing process or died after pairing, it was replaced with another male reared under the same conditions. Once females started ovipositing, egg masses were collected on a daily basis, and the number of eggs was recorded until all adult specimens had died. After the eggs hatched, the number of newly emerged larvae was recorded daily and the hatching rate was then calculated.

### 2.4. Life Table Data Analysis

According to the age-stage, two-sex life table theory [29,30], the TWOSEX-MSChart program was employed to analyze the life history data of *P. nuda* reared on *F. microcarpa* subjected to different levels of drought stress [31]. Including the oviposition period, adult preoviposition period (APOP), total preoviposition period (TPOP), and age-stage survival rate (*s_xj_*) (*x* = age, *j* = stage), representing the probability of newly laid eggs surviving to age *x* and stage *j*, and age-stage-specific fecundity (*f_xj_*), defined as the number of eggs laid by female adults at age *x*. Then, the age-specific survival rate (*l_x_*) is calculated as the probability that a newborn egg survives to age *x*.

$$lx=\sum_{j=1}^{m}s_{xj}$$

*m* is the number of stages.

The age-specific fecundity (*m_x_*) is the average number of eggs laid per individual that survives to age *x*.

$$m_{x}=\frac{\sum_{j=1}^{m}s_{xj}f_{xj}}{\sum_{j=1}^{m}s_{xj}}$$

The net reproductive rate (*R*_0_) is the total number of offspring one female individual can produce throughout its lifetime.

$$R_{0}=\sum_{X=0}^{\infty }I_{x}m_{x}$$

Life expectancy (*e_xj_*) is the remaining survival time of individuals at age *x* and developmental stage *j*.

$$e_{xj}=\sum_{i=x}^{\infty }\sum_{j=y}^{m}s_{iy}^{\prime }$$

The age-stage-specific reproductive value (*v_xj_*) is the contribution of individuals of age *x* and stage *j* to the future population.

$$V_{xj}=\frac{e^{r(x+1)}}{s_{xj}}\sum_{i=x}^{\infty }e^{-r(i+1)}\sum_{y=j}^{m}s_{iy}^{\prime }f_{iy}$$

The intrinsic rate of increase (*r*) is the average daily growth rate of the population when it reaches a stable age distribution.

$$\sum_{x=0}^{\infty }e^{-r(x+1)}I_{x}m_{x}=1$$

The finite rate of increase (*λ*) is the rate of population growth as time approaches infinity and the population reaches a stable age-stage distribution.

$$\lambda =e^{r}$$

The mean generation time (*T*) is the time it takes for a population to increase to *R*_0_ times its size at the stable age stage.

$$T=\frac{\mathrm{In}R_{0}}{r}$$

### 2.5. Population Projection

Based on the life table data generated by TWOSEX-MSChart, population projections were conducted using TIMING-MSChart to assess how the rearing of *P. nuda* on *F. microcarpa* subjected to different levels of drought stress affects its 200-day population dynamics [32].

### 2.6. Statistical Analysis

The biological parameters associated with growth, development, and reproduction of *P. nuda* were analyzed using the TWOSEX-MSChart software across different treatment groups [29]. The mean values and standard errors for each parameter were estimated through 100,000 bootstrap replications [33], and intergroup differences were assessed using paired bootstrap tests [34,35]. One-way ANOVA was employed to compare the differences in pupal weight and egg hatching rate among different treatment groups, and Tukey’s HSD test was utilized for multiple comparisons. The data are presented as the mean ± standard error, and the significance level was set at *p* < 0.05. The analysis was carried out in IBM SPSS Statistics 23 (Chicago, IL, USA). All figures were drawn using SigmaPlot 14.0 (OriginLab Corporation, Northampton, MA, USA).

## 3. Results

### 3.1. Developmental Duration and Lifespan of P. nuda Reared on F. microcarpa Under Different Levels of Drought Stress

*P. nuda* successfully completed its generational development on *F. microcarpa* subjected to three different levels of drought stress. However, being reared on *F. microcarpa* leaves subjected to different levels of drought stress had an impact on the larval development time and adult lifespan. Among these, reared on *F. microcarpa* subjected to light and severe drought stress, respectively, shortened the developmental periods of 4th–7th instar larvae (*p* < 0.05) and 5th–7th instar larvae (*p* < 0.05). Conversely, reared on plants experiencing moderate drought stress significantly prolonged the developmental period of 1st instar larvae (*p* < 0.05) while shortening that of 5th and 7th instar larvae (*p* < 0.05). In addition, being reared on drought-stressed *F. microcarpa* significantly shortened the overall duration of the larval stage (from the 1st to the 7th instar) (*p* < 0.05). The shortest total larval duration was observed under light (29.58 ± 0.21 d) and severe (30.32 ± 0.36 d) drought stress conditions. In terms of total lifespan, *P. nuda* reared on *F. microcarpa* showed the shortest duration under light drought stress (50.52 ± 0.46 d), while that of *P. nuda* reared on control group of *F. microcarpa* was the longest (53.31 ± 0.60 d) (Table 1).

### 3.2. Pupal Characteristics of P. nuda Reared on F. microcarpa Under Different Levels of Drought Stress

**Figure 1:**
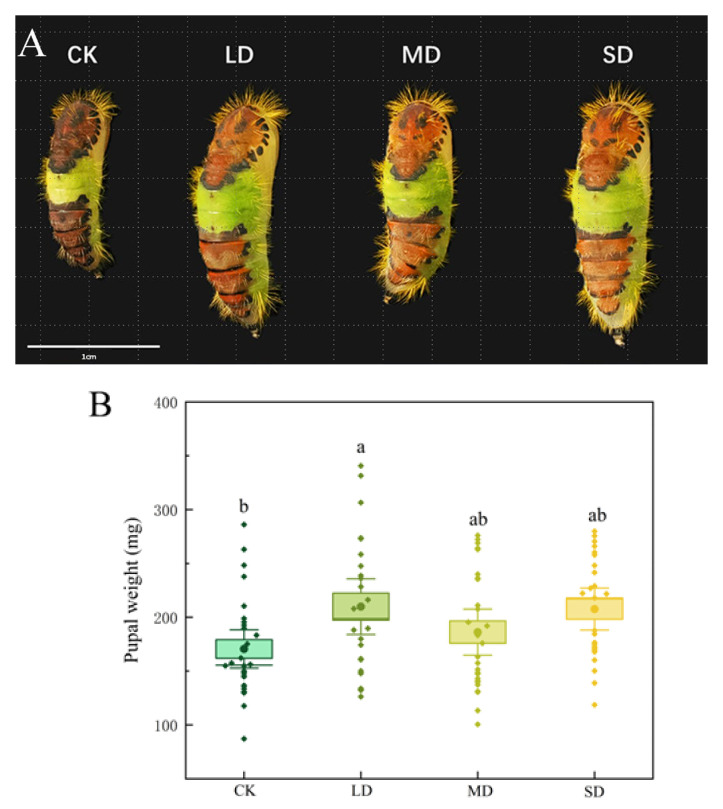
Pupal morphology (**A**) and weight (**B**) of *P. nuda* reared on *F. microcarpa* under different levels of drought stress. Data are mean ± SE and different letters above the bars indicate significant difference (*p* < 0.05).

Reared on *F. microcarpa* plants subjected to drought stress significantly affected the pupal weight of *P. nuda*. Compared with the control group, pupal weight significantly increased under light drought stress (*p* < 0.05). Among all treatments, the pupal weight of *P. nuda* reared on plants subjected to light drought stress was the heaviest (209.88 ± 12.57 mg), whereas the control group had the lightest pupal weight (170.57 ± 8.67 mg), and the former was 23.05% heavier than the latter (Figure 1A,B).

### 3.3. Reproductive Parameters of P. nuda Reared on F. microcarpa Under Different Levels of Drought Stress

In terms of reproductive parameters, when *P. nuda* was reared on *F. microcarpa* subjected to light and severe drought stress, the total pre-oviposition period (TPOP) was significantly shortened (*p* < 0.001). Additionally, after being reared on *F. microcarpa* subjected to light and severe drought stress, the number of eggs laid by a single female *P. nuda* was significantly higher than that of the control group (*p* < 0.001). The highest fecundity was observed in the light drought stress group (181.64 ± 3.61 eggs), which was 1.34 times that of the control group (135.23 ± 4.03 eggs). However, drought stress had no significant effect on the egg-hatching rate of *P. nuda* (*p* = 0.671) (Table 2).

### 3.4. Population Parameters of P. nuda Reared on F. microcarpa Under Different Levels of Drought Stress

Rearing *P. nuda* on *F. microcarpa* subjected to different levels of drought stress significantly influenced the population parameters of *P. nuda*. In all treatment groups, the intrinsic rate of increase (*r*) exceeded 0 and the finite rate of increase (*λ*) exceeded 1, indicating that the population of *P. nuda* can still sustain survival and reproduction after being reared on *F. microcarpa* subjected to drought stress. Compared with the control group, the intrinsic rate of increase (*r*) and finite rate of increase (*λ*) of *P. nuda* reared on *F. microcarpa* subjected to light drought stress were significantly higher (*p* < 0.05). However, no significant differences in *r* and λ values were observed between the control group and those subjected to moderate (*r*:*p* = 0.73; *λ*:*p* = 0.73) or severe drought stress (*r*:*p* = 0.22; *λ*:*p* = 0.22). The mean generation time (*T*) was significantly shorter under light (*p* < 0.001) and severe drought stress (*p* < 0.001) compared to the control group, whereas no significant difference was detected under moderate drought stress (*p* = 0.31) (Table 3).

### 3.5. The Age-Stage Survival Rate (s_xj_) of P. nuda Reared on F. microcarpa Under Different Levels of Drought Stress

**Figure 2:**
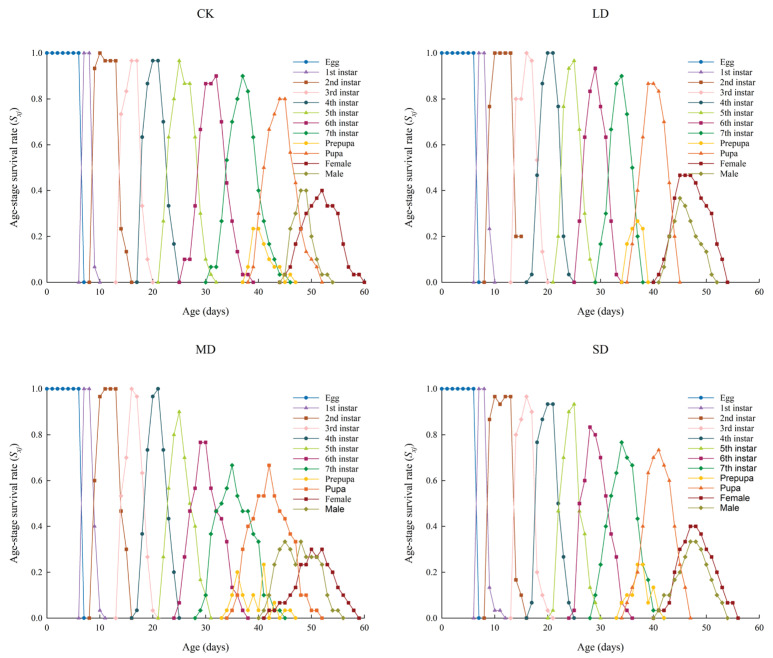
The *s_xj_* values of *P. nuda* reared on *F. microcarpa* under different levels of drought stress.

*P. nuda* was capable of completing its entire generation when reared on *F. microcarpa* leaves under different levels of drought stress. Owing to variations in individual development rates, a substantial overlap was noted among its developmental stages. The highest survival rate from egg to adulthood, reaching 90.00%, was observed under moderate drought stress, while the lowest, at 76.67%, was recorded under severe stress. The survival rate of adult females reached its peak under light drought stress, whereas the male survival rate was highest in the control group. Moreover, female longevity consistently outperformed that of males across all experimental treatments (Figure 2).

### 3.6. Population Survival Rate and Fecundity of P. nuda Reared on F. microcarpa Under Different Levels of Drought Stress

**Figure 3:**
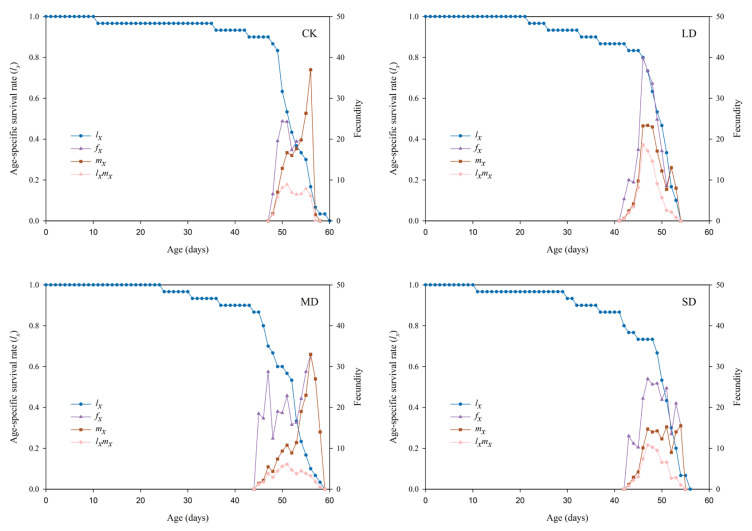
The *l_x_*, *f_x_*, *m_x_*, and *l_x_m_x_* values of *P. nuda* reared on *F. microcarpa* under different levels of drought stress.

The effects of *F. microcarpa* reared under different drought stress treatments on the age-specific survival rate (*l_x_*), age-stage-specific fecundity (*f_xj_*), age-specific fecundity (*m_x_*), and age-stage-specific reproductive value (*l_x_m_x_*) of *P. nuda* are shown in Figure 3. The *l_x_* curves of all treatments showed an initial gradual decline, which was then followed by a more rapid decrease. Final adult mortality occurred earlier in all drought-stressed groups compared to the control group. Specifically, the longest lifespan (60 d) was observed in the control group, in contrast to the shortest (54 d) under light drought stress. The *m_x_* curve indicates that drought stress advanced the initial reproductive time of the population, and this effect was most pronounced under light drought stress. Under light drought stress, the peaks of *f_xj_* and *l_x_m_x_* curve were the highest, while the peak of *m_x_* curve was the highest in the control group.

### 3.7. Life Expectancy (e_xj_) of P. nuda Reared on F. microcarpa Under Different Levels of Drought Stress

**Figure 4:**
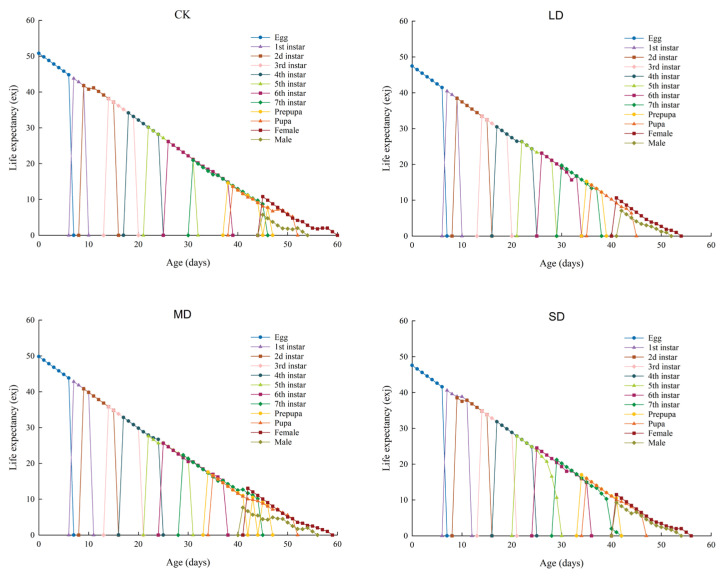
The *e_xj_* values of *P. nuda* reared on *F. microcarpa* under different levels of drought stress.

The life expectancy (*e_xj_*) of *P. nuda* at different treatment groups is shown in Figure 4. Overall, the life expectancy of each treatment group gradually declined to zero with increasing age. Comparisons among different treatments showed that the life expectancy of *P. nuda* individuals reared on *F. microcarpa* without drought stress was the longest (50.8 d), whereas that of the individuals reared on *F. microcarpa* under light drought stress was the shortest (47.47 d). This indicates that the development cycle of the individuals reared on drought-stressed plants was relatively shortened. Moreover, under all treatment conditions, the life expectancy of female adults was longer than that of male adults.

### 3.8. Reproductive Value (v_xj_) of P. nuda Reared on F. microcarpa Under Different Levels of Drought Stress

**Figure 5:**
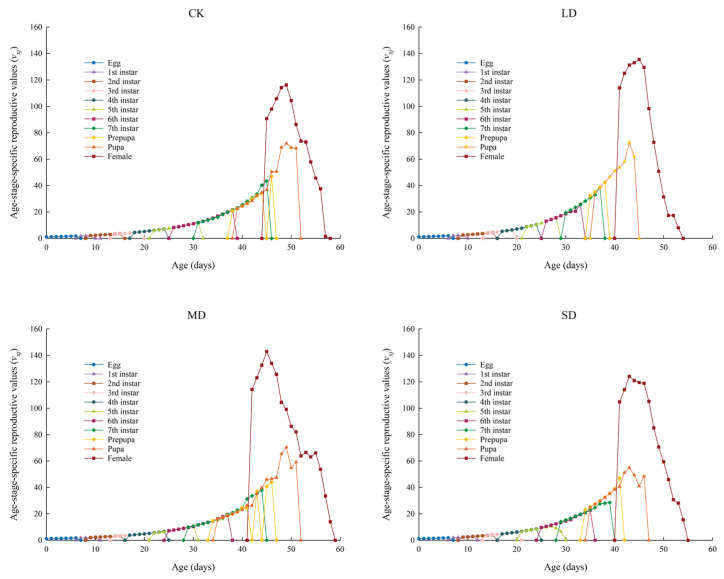
The *v_xj_* values of *P. nuda* reared on *F. microcarpa* under different levels of drought stress.

The *v_xj_* values of *P. nuda* population reared on *F. microcarpa* under different levels of drought stress are shown in Figure 5. The initial *v_xj_* values for *P. nuda* in the control, light, moderate, and severe drought stress groups were 1.08, 1.10, 1.08, and 1.09, respectively. The *v_xj_* values of all groups gradually increased with age and developmental stage, reaching their peaks during the oviposition period of female insects. Among these groups, the moderate drought stress group exhibited the highest peak *v_xj_* value (142.89 eggs), whereas the control group had the lowest (116.20 eggs). The time when all stress groups reached their peaks (light: 45 d; moderate: 45 d; severe: 43 d) was earlier than that of the control group (49 d).

### 3.9. Population Growth Prediction of P. nuda Reared on F. microcarpa Under Different Levels of Drought Stress

**Figure 6:**
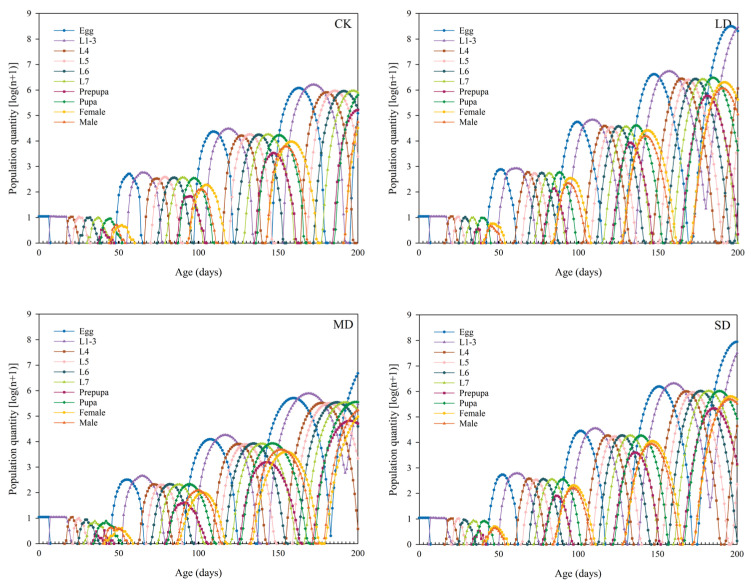
Population projection of *P. nuda* on *F. microcarpa* under different levels of drought stress over 200 days.

TIMEING-MSChart software was used to predict the population dynamics of *P. nuda* reared on *F. microcarpa* under different levels of drought stress in the next 200 days. The results indicate that reared on *F. microcarpa* under drought stress can accelerate the population growth rate of *P. nuda*. Under light and severe drought stress, the population development rate was relatively rapid. By the 200th day, the 5th generation had reached the peak of oviposition, and some larvae had developed to the 5th or 6th instar. In contrast, most individuals in the control group were still in the late larval or pupal stage of the 4th generation. In addition to the disparities in development progress, there were also notable differences in the population sizes among the groups. Specifically, the population sizes of *P. nuda* reared on the leaves of the lightly and severely stressed *F. microcarpa* were significantly larger (Figure 6).

## 4. Discussion

The physiological and biochemical characteristics of host plants play a pivotal role in the growth, development, and reproduction of insects [36]. The physical and chemical properties, as well as the nutritional quality, of host plants have a substantial impact on insect growth rates, survival rates, and reproductive capacity, thus playing a crucial part in determining population dynamics [37]. Our research demonstrates that *P. nuda* can complete its life cycle on *F. microcarpa* under all levels of drought stress. However, there are differences in the responses of *P. nuda* to different levels of drought stress experienced by the host plant, specifically in terms of developmental duration, fecundity, and population parameters. Compared with the control group, larvae of *P. nuda* exhibited a shortened larval development period when reared on drought-stressed *F. microcarpa*. Feeding on lightly or severely drought-stressed *F. microcarpa* led to an increased adult lifespan, a decreased total preoviposition period (TPOP), and a higher fecundity in *P. nuda.* The reduced larval development period of *F. microcarpa* under drought stress might be related to the changes in the plant’s nutritional status and resistance levels triggered by drought stress. Research has indicated that environmental stress can result in alterations in the nutritional components of plant leaves. When insects feed on hosts with lower nutritional value, they typically compensate for nutritional deficiencies by extending the feeding time or increasing food intake [38,39]. Furthermore, within natural ecosystems, a shorter larval development period can curtail the duration of exposure to predators during the immature stages of insects. This reduction in exposure time consequently decreases predation risk and promotes population survival and growth [40]. The findings presented above suggest that drought may indirectly impact the growth, development, and population expansion of *P. nuda* by exerting an influence on their host *F. macrocarpa.*

In addition to the developmental duration, pupal weight is also extensively regarded as a crucial indicator for assessing the suitability of host plants for insects. Heavier pupae typically suggest better adult fitness; thus, pupal weight can effectively mirror the adaptability of herbivorous insects to their host plants [41,42]. The quantity of eggs produced by female insects typically shows a positive correlation with pupal weight. This correlation suggests that heavier pupae are linked to more conducive conditions provided by the host plant for insect growth, reproduction, and population expansion [43,44]. For instance, beet webworm (*Loxostege sticticalis*) individuals that consume pigweed (*Portulaca oleracea*) demonstrate greater pupal weights and higher population growth rates when compared to those reared on agricultural crops like soybean (*Glycine max*) and pea (*Pisum sativum*) [45]. Our research indicates that *F. microcarpa* under drought stress can indirectly affect the pupal weight of *P. nuda*. Larvae reared on *F. microcarpa* under light drought stress conditions result in increased pupal mass and higher fecundity per female. These findings indicate that specific levels of drought stress can enhance the suitability of host plants for *P. nuda*, thus facilitating better growth, development, and population expansion.

Life table parameters (*R*_0_, *r*, *λ*, *T*) are key indicators that characterize the population’s growth potential. These parameters integrate data on survival rates, reproductive capacity, and developmental rates, enabling a sensitive reflection of the combined effects of various environmental and biological factors on overall population fitness [46,47]. Our research indicates that the population of *P. nuda* is capable of completing its entire life cycle under various drought stress conditions. However, the light stress group showed a higher *r* and *λ*, as well as a shorter *T* compared to the control group. Generally, while the fitness of insects on host plants is influenced by multiple factors, it primarily depends on the chemical defense substances and nutritional quality of the plants [48,49]. Previous studies have shown that drought stress can lead to an increase in the concentrations of amino acids and free sugars in tomato (*Solanum lycopersicum*) plants, thereby enhancing their nutritional value for the two spotted spider mite (*Tetranychus urticae*) and promoting the growth of its population [50]. In contrast, under drought stress, the activity of protease inhibitors in soybean (*Glycine max*) plants increases, enhancing their resistance to *Anticarsia gemmatalis* and significantly increasing the mortality rate of the pest’s larvae [51]. In this study, the growth, development, and reproductive performance of *P. nuda* were enhanced when individuals were reared on *F. microcarpa* plants that had been subjected to light and severe drought stress, as compared to the control group. Among these groups, the performance of the light stress group was the best, followed by that of the severe stress group. This can be attributed to the prioritization of plant growth over defense mechanisms under mild drought stress, which leads to increased concentrations of nutrients such as soluble sugars and free amino acids in plants, thus enhancing the nutritional acquisition efficiency of insects [52]. However, when plants experience severe drought stress, their defense systems are impaired because a large amount of resources are reallocated to drought-tolerance mechanisms (e.g., stomatal closure and osmotic adjustment). This results in a notable down-regulation of JA-mediated anti-herbivore defenses, such as protease inhibitors and polyphenol oxidases [53]. Under moderate drought stress, the performance of *P. nuda* exhibited no significant difference compared to the control group. This phenomenon may be ascribed to an elevated carbon-nitrogen ratio in plants, which is conducive to nutrient acquisition for insects. However, this advantage is offset by a concurrent increase in defensive compounds, leading to a dynamic equilibrium between nutrition and defense [54]. In the future, conducting further research on the physiological and molecular response mechanisms of *P. nuda* when reared on hosts exposed to different levels of drought stress will be a meaningful research direction.

Life tables are one of the important tools for assessing the suitability of pests to host plants [55]. However, traditional life table methods frequently fail to precisely reflect the dynamic characteristics of insect populations. This is because they neglect male individuals, overlook differences in developmental stages, and ignore individual variations [23]. Therefore, this study adopted the age-stage, two-sex life table method. This method can simultaneously integrate data from both sexes and effectively overcome the aforementioned limitations, thus offering a more accurate depiction of insect population structure [23]. Our research results indicate that the *s_xj_*, *e_xj_*, and *v_xj_* curves all demonstrate significant stage overlap. This overlap, which can be attributed to variations in individual developmental rates, is commonly observed in insect populations [56]. The overlap of developmental stages enhances the resilience of insect populations against environmental fluctuations. Even if individuals of specific age-groups die off due to changing environmental conditions, the concurrent presence of other developmental stages guarantees continued survival and preserves population continuity [57]. Nevertheless, this phenomenon presents more significant challenges for practitioners in their control endeavors. Furthermore, the peak values of *v_xj_* and *m_x_* were observed in the moderate drought stress treatment and the control group, rather than in the light drought stress treatment that showed the highest egg production. This pattern can be ascribed to the more dispersed oviposition timing under light stress conditions. The results also indicated that female adults consistently had longer lifespans than males in all treatment groups. This advantage of female longevity probably enhances the reproductive success of the population by enabling an extended dispersal period to find suitable oviposition sites [58]. Further predictions of the population dynamics over a 200-day period indicated that *P. nuda* reared on *F. microcarpa* subjected to light and severe drought stress might experience an increase in its population growth rate, thereby leading to an expansion of its population size. This finding corroborates the hypothesis that drought stress may enhance the adaptability of *P. nuda* to *F. microcarpa*. From the perspective of the host plant, it explains the outbreak of herbivorous insect populations in years with less precipitation [59].

This study systematically evaluated the comprehensive impact of rearing *F. microcarpa* under different levels of drought stress on the fitness of the *P. nuda* population. The results demonstrated that the larval development duration was significantly reduced under all drought stress conditions compared to the control group. Under both light and severe drought stress, adult lifespan was prolonged, the total preoviposition period (TPOP) was shortened, fecundity per female increased, and generation time (*T*) was significantly reduced. Furthermore, pupal weight, intrinsic rate of increase (*r*), and finite rate of increase (*λ*) were significantly enhanced, but only under light drought stress. Age-stage, two-sex life table analysis further demonstrated that under drought stress conditions, the population showed a decreased life expectancy and an earlier reproductive peak, which in turn affected the population dynamics of *P. nuda* over a 200-day period. Based on these results, it is advisable to enhance the irrigation frequency for landscape tree species like *F. microcarpa* during periods of low rainfall to reduce the risk of pest population outbreaks caused by drought-induced stress in host plants.

## 5. Conclusions

The findings of this study demonstrate that rearing *P. nuda* on *F. microcarpa* subjected to drought stress significantly affects its growth, development, and reproductive performance. The extent of this effect depends on the intensity of drought stress. Different levels of drought stress differentially influence these biological processes by inducing changes in the physiological status of the host plant, showing a nonlinear response pattern. Under light drought conditions, fitness reaches its peak, leading to optimal performance in developmental, reproductive, and population growth parameters. The impact of moderate drought was negligible, and there were no significant differences in various parameters when compared with the control group. Although severe drought still enhances certain life-history traits, its effects are relatively weaker than those under light drought stress. The population prediction results also indicate that the population size grows more rapidly and is larger under light and severe drought conditions. This study conducted a systematic evaluation of the effects of varying levels of drought stress on the feeding adaptability of *F. microcarpa* to *P. nuda*. Future research can incorporate a broader range of experimental conditions and more diverse methodologies to further elucidate the influence of drought stress on the population dynamics of *Ficus* plants and other plant groups, as well as their associated herbivorous pests. The findings facilitate a more in-depth understanding of how drought stress affects the population dynamics of herbivorous insects under the backdrop of global climate change and offer a scientific basis for formulating a scientific and precise pest control strategy.

## Figures and Tables

**Table 1:** The developmental duration and lifespan of *P. nuda* reared on *F. microcarpa* under different levels of drought stress.

Development Stage (d)	N	CK	N	LD	N	MD	N	SD
Egg	30	7.00 ± 0.00 ^a^	30	7.00 ± 0.00 ^a^	30	7.00 ± 0.00 ^a^	30	7.00 ± 0.00 ^a^
1st instar	30	2.07 ± 0.05 ^b^	30	2.23 ± 0.08 ^ab^	30	2.43 ± 0.10 ^a^	30	2.20 ± 0.11 ^ab^
2nd instar	29	5.31 ± 0.11 ^a^	30	5.17 ± 0.13 ^a^	30	5.33 ± 0.12 ^a^	29	5.07 ± 0.07 ^a^
3rd instar	29	4.07 ± 0.08 ^a^	30	4.23 ± 0.13 ^a^	30	4.13 ± 0.11 ^a^	29	4.00 ± 0.07 ^a^
4th instar	29	4.79 ± 0.16 ^a^	29	4.38 ± 0.12 ^b^	30	4.47 ± 0.15 ^ab^	29	4.55 ± 0.16 ^ab^
5th instar	29	5.66 ± 0.18 ^a^	28	4.11 ± 0.09 ^b^	29	4.48 ± 0.20 ^b^	28	4.14 ± 0.16 ^b^
6th instar	29	5.62 ± 0.17 ^a^	27	4.59 ± 0.12 ^c^	28	5.11 ± 0.20 ^ab^	27	5.04 ± 0.21 ^bc^
7th instar	28	6.04 ± 0.15 ^a^	26	4.81 ± 0.17 ^c^	27	5.48 ± 0.16 ^b^	25	5.36 ± 0.19 ^b^
1st to 7th instar	28	33.68 ± 0.37 ^a^	26	29.58 ± 0.21 ^c^	27	31.63 ± 0.57 ^b^	25	30.32 ± 0.36 ^c^
Pre-pupa	28	1.04 ± 0.03 ^a^	26	1.04 ± 0.04 ^a^	27	1.07 ± 0.05 ^a^	25	1.16 ± 0.07 ^a^
Pupa	26	6.00 ± 0.05 ^a^	25	5.92 ± 0.05 ^a^	27	6.00 ± 0.07 ^a^	23	6.00 ± 0.06 ^a^
Adult	26	5.65 ± 0.39 ^b^	25	7.00 ± 0.36 ^a^	27	6.22 ± 0.36 ^ab^	23	7.26 ± 0.39 ^a^
Total longevity	26	53.31 ± 0.60 ^a^	25	50.52 ± 0.46 ^b^	27	51.93 ± 0.76 ^ab^	23	51.78 ± 0.51 ^ab^

Note: The data are mean ± SE, and different letters in the same row indicate significant differences (*p* < 0.05). CK: normal water supply, LD: light drought stress, MD: moderate drought stress, SD: severe drought stress; N: numbers of individual *P. nuda* that completed their development, the same below.

**Table 2:** The reproductive parameters of *P. nuda* reared on *F. microcarpa* under different levels of drought stress.

Reproductive Parameters	Water Regime			
	CK	LD	MD	SD
Oviposition days (d)	4.69 ± 0.38 ^a^	5.57 ± 0.34 ^a^	4.90 ± 0.31 ^a^	5.42 ± 0.28 ^a^
APOP (d)	2.15 ± 0.27 ^a^	1.71 ± 0.16 ^a^	2.30 ± 0.33 ^a^	1.67 ± 0.14 ^a^
TPOP (d)	50.69 ± 0.60 ^a^	45.21 ± 0.40 ^b^	49.80 ± 0.98 ^a^	46.25 ± 0.50 ^b^
Fecundity (number of egg)	135.23 ± 4.03 ^c^	181.64 ± 3.61 ^a^	144.60 ± 3.77 ^c^	159.58 ± 4.33 ^b^
Hatching rate (%)	88.87 ± 1.54 ^a^	89.33 ± 1.82 ^a^	91.81 ± 1.17 ^a^	89.52 ± 1.95 ^a^

Note: The data are mean ± SE, and different letters in the same row indicate significant differences (*p* < 0.05).

**Table 3:** The population parameters of *P. nuda* reared on *F. microcarpa* under different levels of drought stress.

Population Parameters	Water Regime			
	CK	LD	MD	SD
Net reproductive rate (*R*_0_)	58.60 ± 12.39 ^a^	84.77 ± 16.60 ^a^	48.20 ± 12.48 ^a^	63.83 ± 14.40 ^a^
Intrinsic rate of increase (*r*/day)	0.0766 ± 0.0044 ^b^	0.0924 ± 0.0044 ^a^	0.0746 ± 0.0056 ^b^	0.0845 ± 0.0050 ^ab^
Finite rate of increase (*λ*/day)	1.08 ± 0.00 ^b^	1.10 ± 0.00 ^a^	1.08 ± 0.01 ^b^	1.09 ± 0.01 ^ab^
Mean generation time (*T*/day)	53.13 ± 0.58 ^a^	48.05 ± 0.45 ^b^	51.98 ± 0.98 ^a^	49.16 ± 0.60 ^b^

Note: The data are mean ± SE, and different letters in the same row indicate significant differences (*p* < 0.05).

## Data Availability

The original contributions presented in this study are included in the article. Further inquiries can be directed to the corresponding author.

## References

[1] Peng S.M., Jiang H., Zhang S., Chen L.H., Li X.G., Korpelainen H., Li C.Y. (2012). Transcriptional profiling reveals sexual differences of the leaf transcriptomes in response to drought stress in *Populus yunnanensis*. Tree Physiol..

[2] Ault T.R. (2020). On the essentials of drought in a changing climate. Science.

[3] Yang X.D., Wu N.C., Gong X.W. (2023). Plant adaptation to extreme environments in drylands. Forests.

[4] Li C.J., Fu B.J., Wang S., Stringer L.C., Wang Y.P., Li Z.D., Liu Y.X., Zhou W.X. (2021). Drivers and impacts of changes in China’s drylands. Nat. Rev. Earth Environ..

[5] Bouabdelli S., Zeroual A., Meddi M., Assani A. (2022). Impact of temperature on agricultural drought occurrence under the effects of climate change. Theor. Appl. Climatol..

[6] Markus M., Cai X.M., Sriver R. (2019). Extreme floods and droughts under future climate scenarios. Water.

[7] Atkinson N.J., Urwin P.E. (2012). The interaction of plant biotic and abiotic stresses: From genes to the field. J. Exp. Bot..

[8] He X.S., Xu L.C., Pan C., Gong C., Wang Y.J., Liu X.L., Yu Y.C. (2020). Drought resistance of *Camellia oleifera* under drought stress: Changes in physiology and growth characteristics. PLoS ONE.

[9] Suzuki N., Rivero R.M., Shulaev V., Blumwald E., Mittler R. (2014). Abiotic and biotic stress combinations. New Phytol..

[10] Mundim F.M., Vieira-Neto E.H.M., Alborn H., Bruna E.M. (2021). Disentangling the influence of water limitation and simultaneous above and belowground herbivory on plant tolerance and resistance to stress. J. Ecol..

[11] Zhang K.X., Li H.Y., Quandahor P., Gou Y.P., Li C.C., Zhang Q.Y., Haq I.U., Ma Y., Liu C.Z. (2022). Responses of six wheat cultivars (*Triticum aestivum*) to wheat aphid (*Sitobion avenae*) infestation. Insects.

[12] Zhang K.X., Ma Y., Li C.C., Quandahor P., Haq I.U., Zhang Q.Y., Kong L.L., Tao Y., Liu C.Z. (2023). Population growth of *Tetranychus truncatus* (Acari: Tetranychidae) on different drought-tolerant potato cultivars. J. Econ. Entomol..

[13] Kansman J.T., Basu S., Casteel C.L., Crowder D.W., Lee B.W., Nihranz C.T., Finke D.L. (2022). Plant water stress reduces aphid performance: Exploring mechanisms driven by water stress intensity. Front. Ecol. Evol..

[14] Cui H.Y., Wang L.Y., Reddy G.V.P., Zhao Z.H. (2021). Light drought facilitates the increase in wheat aphid abundance by changing host metabolism. Ann. Entomol. Soc. Am..

[15] Zhang K.X., Lin C.Y., Liu H.P., Haq I.U., Quandahor P., Gou Y.P., Li C.C., Yang Z.Y., Liu C.Z. (2024). Drought reduced the adaptability of *Myzus persicae* on drought-tolerant potato cultivars. Entomol. Gen..

[16] Cottee-Jones H.E.W., Bajpai O., Chaudhary L.B., Whittaker R.J. (2016). The Importance of *Ficus* (Moraceae) trees for tropical forest restoration. Biotropica.

[17] Hussain A., Tian M.Y., He Y.R., Ahmed S. (2009). Entomopathogenic fungi disturbed the larval growth and feeding performance of *Ocinara varians* (Lepidoptera: Bombycidae) larvae. Insect Sci..

[18] Zheng X.L., Liu J.Y., Zhang Z.L., Wang P., Lu W. (2019). Diel rhythms of sexual behavior and pheromone responses in *Phauda flammans* Walker (Lepidoptera: Zygaenidae). Pest. Manag. Sci..

[19] Liao S.K., Lin H.Y., Wang J.J., Wang Q., Wei H.J., Chen H. (2024). Effects of different *Ficus* feeding experiences on host preference of *Perina nuda* larvae (Lepidoptera: Lymantriidae). J. Econ. Entomol..

[20] Mao X.J., Zheng H.S., Liao S.K., Wei H.J., Lin H.Y., Wang Q., Chen H. (2024). Predicting the potential distribution of *Perina nuda* under climate change in China. Entomol. Exp. Appl..

[21] Wakamura S., Arakaki N., Yamazawa H., Nakajima N., Yamamoto M., Ando T. (2002). Identification of epoxyhenicosadiene and novel diepoxy derivatives as sex pheromone components of the clear-winged tussock moth *Perina nuda*. J. Chem. Ecol..

[22] Liao S.K., Huang J.H., Lin H.Y., Wang Q., Wang J.J., Mao X.J., Wei H.J., Chen H. (2024). Effects of temperature stress on demographic traits and population projection of *Perina nuda* (Lepidoptera: Lymantriidae). J. Asia-Pac. Entomol..

[23] Chi H., You M.S., Atlıhan R.M., Smith C.L., Kavousi A., Özgökçe M.S., Güncan A., Tuan S.J., Fu J.W., Xu Y.Y. (2020). Age-Stage, two-sex life table: An introduction to theory, data analysis, and application. Entomol. Gen..

[24] Ullah F., Haq I.U., Gul H., Hafeez M., Güncan A., Tariq K., Desneux N., Zhao Z.H., Li Z.H. (2022). Impact of temperature stress on demographic traits and population projection of *Bactrocera dorsalis*. Entomol. Gen..

[25] Chi H., Kavousi A., Gharekhani G., Atlihan R., Salih Özgökçe M., Güncan A., Gökçe A., Smith C.L., Benelli G., Guedes R.N.C. (2023). Advances in theory, data analysis, and application of the age-stage, two-sex life table for demographic research, biological control, and pest management. Entomol. Gen..

[26] Saska P., Skuhrovec J., Platková H., Kosová K., Tylová E., Tuan S.J., Vítámvás P. (2023). Response of the spring wheat-cereal aphid system to drought: Support for the plant vigour hypothesis. J. Pest Sci..

[27] Zhang X.Y., Liu W.T., Lv Y.C., Li T.L., Tang J.Z., Yang X.H., Bai J., Jin X., Zhou H.T. (2022). Effects of drought stress during critical periods on the photosynthetic characteristics and production performance of Naked oat (*Avena nuda* L.). Sci. Rep..

[28] He F., Wu Z.Q., Zhao Z.B., Chen G., Wang X.G., Cui X.L., Zhu T.H., Chen L.H., Yang P., Bi L.F. (2022). Drought stress drives sex-specific differences in plant resistance against herbivores between male and female poplars through changes in transcriptional and metabolic profiles. Sci. Total Environ..

[29] Chi H., Liu H. (1985). Two new methods for the study of insect population ecology. Bull. Inst. Zool. Acad. Sin..

[30] Chi H. (1988). Life-table analysis incorporating both sexes and variable development rates among individuals. Environ. Entomol..

[31] Chi H. (2025). TWOSEX-MSChart: A Computer Program for the Age-Stage, Two-Sex Life Table Analysis.

[32] Chi H. (2025). TIMING-MSChart: A Computer Program for the Population Projection Based on Age-Stage, Two-Sex Life Table.

[33] Huang H.W., Chi H., Smith C.L. (2018). Linking demography and consumption of *Henosepilachna vigintioctopunctata* (Coleoptera: Coccinellidae) fed on *Solanum photeinocarpum* (Solanales: Solanaceae): With a new method to project the uncertainty of population growth and consumption. J. Econ. Entomol..

[34] Xie W., Zhi J.R., Ye J.Q., Zhou Y.M., Li C., Liang Y.J., Yue W.B., Li D.Y., Zeng G., Hu C.X. (2021). Age-stage, two-sex life table analysis of *Spodoptera frugiperda* (JE Smith) (Lepidoptera: Noctuidae) reared on maize and kidney bean. Chem. Biol. Technol. Agric..

[35] Akköprü E.P., Atlıhan R., Okut H., Chi H. (2015). Demographic assessment of plant cultivar resistance to insect pests: A case study of the dusky-veined walnut aphid (Hemiptera: Callaphididae) on five walnut cultivars. J. Econ. Entomol..

[36] La Rossa F.R., Vasicek A., López M.C. (2013). Effects of pepper (*Capsicum annuum*) cultivars on the biology and life table parameters of *Myzus persicae* (Sulz.) (Hemiptera: Aphididae). Neotrop. Entomol..

[37] Obopile M., Ositile B. (2010). Life table and population parameters of cowpea aphid, *Aphis craccivora* Koch (Homoptera: Aphididae) on five cowpea *Vigna unguiculata* (L. Walp.) cultivars. J. Pest Sci..

[38] Behmer S.T. (2009). Insect herbivore nutrient regulation. Annu. Rev. Entomol..

[39] da Silva D.M., Bueno A.F., Andrade K., Stecca C.S., Neves P.M.O.J., Oliveira M.C.N. (2017). Biology and nutrition of *Spodoptera frugiperda* (Lepidoptera: Noctuidae) fed on different food sources. Sci. Agric..

[40] Cisternas M.F., Ríos R.S., Gianoli E. (2022). Gregarious caterpillars shorten their larval development time in response to simulated predation threat. Ecol. Entomol..

[41] Miller W.E. (2005). Extrinsic effects on fecundity-maternal weight relations in capital-breeding Lepidoptera. J. Lepid. Soc..

[42] Eck D.J., Shaw R.G., Geyer C.J., Kingsolver J.G. (2015). An integrated analysis of phenotypic selection on insect body size and development time. Evolution.

[43] Takahashi C.G., Kalns L.L., Bernal J.S. (2012). Plant defense against fall armyworm in micro-sympatric maize (*Zea mays* ssp. *mays*) and Balsas teosinte (*Zea mays* ssp. *parviglumis*). Entomol. Exp. Appl..

[44] Juárez M.L., Schöfl G., Vera M.T., Vilardi J.C., Murúa M.G., Willink E., Hänniger S., Heckel D.G., Groot A.T. (2014). Population structure of *Spodoptera frugiperda* maize and rice host forms in South America: Are they host strains?. Entomol. Exp. Appl..

[45] Ji X.W., Jiang X.F., Yin J., Chen J.L., Ding T.B., Tan X.L. (2024). The adaptability of beet webworm (*Loxostege sticticalis*) to soybeans and other different host plants. Agronomy.

[46] Awmack C.S., Leather S.R. (2002). Host plant quality and fecundity in herbivorous insects. Annu. Rev. Entomol..

[47] Wei M.F., Chi H., Guo Y.F., Li X.W., Zhao L.L., Ma R.Y. (2020). Demography of *Cacopsylla chinensis* (Hemiptera: Psyllidae) reared on four cultivars of *Pyrus bretschneideri* (Rosales: Rosaceae) and *P. communis* pears with estimations of confidence intervals of specific life table statistics. J. Econ. Entomol..

[48] Ulusoy M.R., Ölmez-Bayhan S. (2006). Effect of certain brassica plants on biology of the cabbage aphid *Brevicoryne brassicae* under laboratory conditions. Phytoparasitica.

[49] Zarghami S., Allahyari H., Bagheri M.R., Saboori A. (2010). Effect of nitrogen fertilization on life table parameters and population growth of *Brevicoryne brassicae*. Bull. Insectology.

[50] Ximénez-Embún M.G., Castañera P., Ortego F. (2017). Drought stress in tomato increases the performance of adapted and non-adapted strains of *Tetrachycus urticae*. J. Insect Physiol..

[51] Faustino V.A., de Almeida Barros R., da Silva Júnior N.R., Barbosa S.L., Vital C.E., da Silva F.L., Cabrera Y.B.M., Campos W.G., de Oliveira Ramos H.G., de Almeida Oliveira M.G. (2021). Soybean drought-stressed plants impair *Anticarsia gemmatalis* (Lepidoptera: Erebidae) midgut proteolytic activity and survival. Phytoparasitica.

[52] He Z.H., Webster S., He S.Y. (2022). Growth-defense trade-offs in plants. Curr. Biol..

[53] Schultz J.C., Appel H.M., Ferrieri A.P., Arnold T.M. (2013). Flexible resource allocation during plant defense responses. Front. Plant Sci..

[54] Huot B., Yao J., Montgomery B.L., He S.Y. (2014). Growth–defense tradeoffs in plants: A balancing act to optimize fitness. Mol. Plant.

[55] Atlihan R., Kasap I., Özgökçe M.S., Polat-Akköprü E., Chi H. (2017). Population growth of *Dysaphis pyri* (Hemiptera: Aphididae) on different pear cultivars with discussion on curve fitting in life table studies. J. Econ. Entomol..

[56] Li X.F., Feng D.D., Xue Q.Q., Meng T.L., Ma R.Y., Deng A., Chi H., Wu Z.Y., Atlıhan R., Men L.N. (2019). Density-dependent demography and mass-rearing of *Carposina sasakii* (Lepidoptera: Carposinidae) incorporating life table variability. J. Econ. Entomol..

[57] Becklin S., Jin Y., Rebarber R., Tenhumberg B. (2025). Spatial dynamics of a pest population with stage-structure and control. J. Math. Biol..

[58] Pointer M.D., Spurgin L.G., McMullan M., Butler S., Richardson D.S. (2024). Life history correlations and trade-offs resulting from selection for dispersal in *Tribolium castaneum*. J. Evol. Biol..

[59] Zhao C.T., Tian D., Mo Y.E., Yu G., Ji C.J., Fan D.Y., Wang X.P., Fang J.Y. (2025). Trouble follows the needy: More severe leaf herbivory in the resource-poor temperate oak forest than in the birch forest. Funct. Ecol..

